# Multivariate learning framework for long‐term adaptation in the artificial pancreas

**DOI:** 10.1002/btm2.10119

**Published:** 2018-11-12

**Authors:** Dawei Shi, Eyal Dassau, Francis J. Doyle

**Affiliations:** ^1^ Harvard John A. Paulson School of Engineering and Applied Sciences, Harvard University Cambridge MA 02138

**Keywords:** artificial pancreas, data‐driven algorithms, parameter adaptation, safety‐critical control

## Abstract

The long‐term use of the artificial pancreas (AP) requires an automated insulin delivery algorithm that can learn and adapt with the growth, development, and lifestyle changes of patients. In this work, we introduce a data‐driven AP adaptation method for improved glucose management in a home environment. A two‐phase Bayesian optimization assisted parameter learning algorithm is proposed to adapt basal and carbohydrate‐ratio profile, and key feedback control parameters. The method is evaluated on the basis of the 111‐adult cohort of the FDA‐accepted UVA/Padova type 1 diabetes mellitus simulator through three scenarios with lifestyle disturbances and incorrect initial parameters. For all the scenarios, the proposed method is able to robustly adapt AP parameters for improved glycemic regulation performance in terms of percent time in the euglycemic range [70, 180] mg/dl without causing risk of hypoglycemia in terms of percent time below 70 mg/dl.

## 1 INTRODUCTION

For several decades, engineers and clinicians have been working on artificial pancreas (AP) systems to achieve automated blood glucose (BG) regulation for patients with Type 1 diabetes mellitus (T1DM). With the encouraging outcome of recent large‐scale/long‐duration outpatient clinical trials,^1^, ^2^, ^3^, ^4^ current AP systems require user intervention around meals and exercise. Since clinical factors such as basal rate (BR) and carbohydrate ratio (CR) are not fixed but do change for patients with Type 1 diabetes, adaptation and learning are needed and expected from AP systems to improve outcome and enable better control as insulin sensitivity changes. In addition, the effects of physical activity on glucose utilization, production, insulin sensitivity, and absorption strongly depend on its timing, type, and intensity,^5^, ^6^ which vary with lifestyle changes. This forms another motivation to incorporate adaptation in AP design.

To achieve this goal, different approaches to AP adaptation have been investigated in the literature. Automated BR modulation was considered in recent outpatient clinical trials^1^, ^2^ and the first U.S. Food and Drug Administration (FDA) approved commercial hybrid AP system in the United States.^3^ With different degrees of automation and clinician effort, pump setting adaptation was also considered in multiple clinical studies,^7^, ^8^, ^9^ although the detailed techniques were not explicitly reported. In a 12‐week outpatient study,^4^ an automated algorithm monitored by study physicians was employed to perform joint adaptation of BR and CR. In earlier investigations,^10^, ^11^ a run‐to‐run approach was proposed to update BR and meal bolus sizes based on sparse BG measurements; this approach was recently revisited in Reference 12 to adapt CR profile during the day and BR at night based on continuous glucose monitor (CGM) measurements, and was verified through multiweek simulations that took account of insulin sensitivity circadian rhythm. A closely related approach that can exploit CGM measurements and continuous insulin delivery is iterative learning control, which can be regarded as a two‐timescale enhancement of run‐to‐run methods.^13^ In Reference 14, iterative learning model predictive control (MPC) was proposed to adapt the reference trajectory of the closed‐loop controller used for glucose regulation, which was tested in a pilot study recently.^15^

Despite the aforementioned progress, the problem of systematically adapting multiple key AP parameters (e.g., segments in BR and CR profiles, controller parameters) under lifestyle disturbances while explicitly enforcing safety considerations remains to be explored. In this work, a two‐phase data‐driven multivariate parameter learning framework for long‐term adaptation of AP is developed. The proposed learning algorithm features a controller‐led approach to adaptation of BR profile and a Bayesian optimization (BO) approach to adaptation of the CR profile and the controller parameters. The effectiveness of the algorithm is evaluated using the entire 111‐adult cohort of the FDA‐accepted Universities of Virginia (UVA)/Padova T1DM simulator^16^ for multiple challenging scenarios. The obtained results suggest that the proposed AP adaptation method can robustly improve glucose regulation performance in terms of percent time in the euglycemic range and average glucose levels without causing the risk of hypoglycemia. A related topic to AP adaptation is individualized AP design, the key idea of which is to design the AP algorithms based on patient‐specific parameters or historical data for improved glucose regulation performance.^17^, ^18^ Generally, AP adaptation attempts to *dynamically* adjust the individualized parameters (e.g., BR and CR profiles) that might have been improperly determined or change slowly in time, while AP individualization features more on static personalization of glucose control performance based on individualized parameters or personal patient data, which can be regarded as AP adaptation if the individualization procedures are performed progressively in time. In addition, AP adaptation can work seamlessly on top of an individualized controller; in our work, the lower‐level MPC algorithm adopts a control‐relevant model personalized by total daily insulin.

## 2 METHODS AND MATERIALS

Patients with T1DM suffer from malfunctions of the glucose metabolic system due to the failure of the pancreas to secrete insulin and require external insulin infusion to regulate excessive BG. An AP attempts to automate glucose regulation through generating insulin micro‐boluses according to real time BG measurements. To assist with the home use of an AP, a dual‐layer control system scheme is first introduced. In the following, the parameter adaptation problem is described and the outline of the solution proposed is discussed.

The human glucose metabolic process is affected by disturbances that evolve under different time scales, including (a) food intake, physical exercise, stress, and alcohol consumption occurring on a random basis within a day, (b) the diurnal circadian rhythm of the body sensitivity to insulin and life habits that repeat on a daily/weekly basis, (c) randomly occurring sickness that can affect insulin sensitivity for one or a couple of days (e.g., influenza and gastrointestinal disease), and (d) chronic metabolic variations due to aging and lifestyle change. To achieve satisfactory glucose regulation performance, these disturbances need to be considered in the design of AP algorithms.

**Figure 1:**
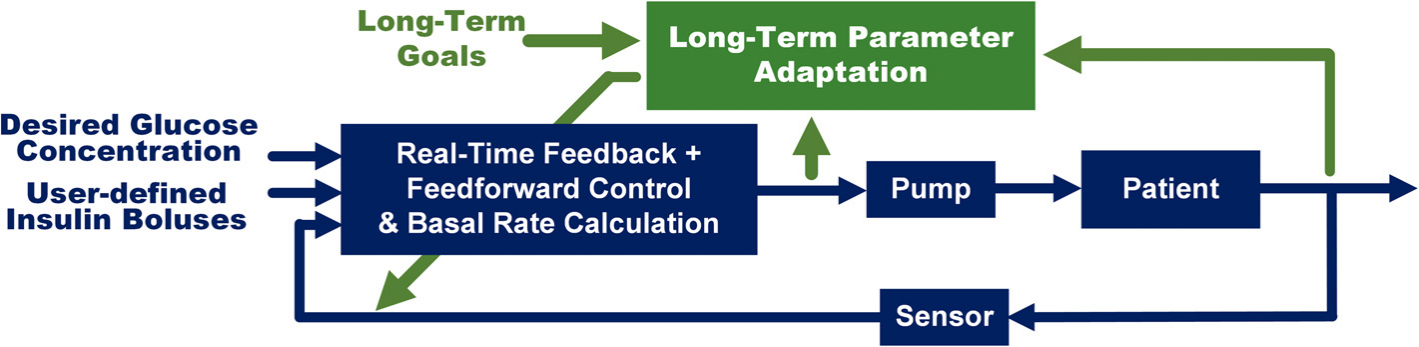
Dual‐layer control scheme. The lower layer (highlighted in blue color) deals with disturbances occurring on smaller time scales based on real time CGM data, while the upper layer (highlighted in green color) handles chronic changes in the glucose metabolic process based on recent performance metrics from the lower layer

Considering the multi‐timescale nature of the disturbances, we introduce a dual‐layer control scheme in this work (see Figure 1). The lower layer is composed of multiple controllers that deal with short‐term disturbances, including

1. a feedforward BR calculator that provides the basal insulin delivery rate based on a predetermined piecewise constant BR profile to counteract against patient's daily life habit and the diurnal insulin sensitivity.
2. a feedforward meal controller that calculates reference meal and correction boluses based on meal information and additional insulin requests provided by the patient.
3. a feedback controller that deals with all uncompensated disturbances based on real‐time CGM measurements and safety considerations.

The feedback controller tops up the BR doses provided by the BR calculator to finalize real‐time insulin microboluses. With a properly designed BR profile, the feedback controller does not need to act with abruptly varying and scenario‐dependent aggressiveness, which reduces the difficulty of feedback control design and the chance of faulty controller‐induced overdose behavior. Feedforward control (e.g., meal boluses) can reduce high‐glucose events and is therefore important in glucose management, particularly when meals cannot be accurately predicted. In the AP literature, lower‐layer control system design has been extensively investigated, the focus of which has been developing safe and efficient feedback control algorithms to improve glucose management; interested readers are referred to Reference 19 for a detailed review of control design for the AP. In our algorithm, we assume that lower layer control tasks are handled by a periodic zone MPC with asymmetric costs^20^ together with the meal bolus strategy introduced therein, although the proposed approach can be applied to other lower‐layer control algorithms.

As is shown in Figure 1, the upper layer is responsible for long‐term parameter adaptation of lower layer control algorithms. This layer evolves on a longer timescale (e.g., weeks) and handles chronic changes in the patient's glucose metabolic process and lifestyle through long‐term AP adaptation algorithms, based on historical performance metric records from the lower layer. With the successful deployment of large and long‐term out‐patient clinical studies, long‐term AP adaptation has gained in importance only recently, and is the main focus of this article.

With the dual‐layer control scheme, we are now ready to introduce the parameter adaptation problem to be investigated. Specifically, the parameters to be adapted include

1. segments {*β*_*n*_} that compose the BR profile *β* ≔ [*β*_1_, *β*_2_, …, *β*_*N*_] used in the BR calculator. Note that each *β*_*n*_ is associated with a time period $T_{n}^{\beta }$ during which it is active. In this work, we assume that $T_{n}^{\beta }$ is fixed and predetermined by clinicians.
2. segments {*γ*_*n*_} that compose the CR profile *γ* ≔ [*γ*_1_, *γ*_2_, …, *γ*_*M*_] used in the meal controller. Similar to *β*, each segment *γ*_*n*_ is used for a particular time period $T_{n}^{\gamma }$, which is assumed to be fixed by clinicians as well.
3. parameters in the closed‐loop controller. Three parameters in the zone MPC developed in Reference 20 are considered, including $\hat{R}$ that represents the control penalty parameter for insulin delivery above the BR, $\bar{D}$ that determines the upper bound of a glucose zone for which the velocity penalty is active, and a coefficient *γ*_IOB_ that determines the responsiveness of the insulin‐on‐board (IOB) constraint.

The goal of this work is to develop an automatic parameter learning algorithm that can correctly adapt the parameters in the lower‐layer control algorithms and is robust to lifestyle disturbances, with minimal patient/clinician involvement and without causing risks of hypoglycemia during the adaptation procedure.

To achieve this goal, a data‐driven multivariate parameter learning framework in proposed. Considering the different roles of the lower‐layer control algorithms, the adaptation procedure is divided into two phases. In Phase I, the parameters in the feedforward control algorithms (namely, the BR and CR profiles) are optimized. Based on the obtained/updated BR and CR profiles, parameters in the feedback control algorithms are adjusted in Phase II. In both phases, we consider the challenging but realistic case that the patient is under closed‐loop control, such that the “open‐loop” parameters are adjusted in Phase I based on closed‐loop data. The detailed adaptation algorithms are presented in Sections 2.1 and 2.2, respectively.

### 2.1 Learning feedforward control parameters

In this subsection, we focus on adapting the parameters in the BR and CR profiles, which is Phase I of the adaptation procedure. The aim here is to obtain reasonable rather than optimal profiles, such that an appropriate operating point is provided for the feedback controller, which helps enhance the safety of closed‐loop glucose control. More importantly, considering the fact that the feedback control may not be available for some periods (e.g., when the controller runs out of battery or lost CGM connection), the obtained parameter needs to be “safe” in the sense that no hypoglycemia would be caused when the patient loses closed‐loop control. In the following, we first separately introduce the methods for BR and CR adaptation, based on which a hybrid time‐ and event‐triggered method is introduced to iterate the procedures that adapt BR and CR.

#### 2.1.1 Adaptation of BR profile

Intuitively, the BR profile *β* is designed to manage “healthy” fasting glucose levels without considering meal‐induced glucose excursions. However, when the fasting glucose levels (measured as glucose levels at night or before meals) are not satisfactory, it is difficult to diagnose which segment *β*_*n*_ in *β* is the root cause. This problem becomes more complicated when the effects of meal boluses are considered, due to a further loss of “identifiability.”^21^ A helpful observation, however, is that the “effective” real‐time BR is ultimately determined by the feedback controller, which is able to adjust a potentially inappropriate BR provided by the BR profile to a certain extent. This observation allows us to perform BR profile adaptation using a controller‐led approach, without the need of explicitly performing root cause diagnosis. To reduce the risk of controller‐induced hypoglycemia, safety constraints are designed to eliminate the adoption of aggressive BR profiles. Through controller‐led BR adaptation, the problem of lack of identifiability/diagnosability can be automatically solved, which leads to a direct separation of the adaptation of BR and CR profiles. The difficulty of algorithm design and implementation, however, is independent of the number of segments in the BR profile.

##### Controller‐led BR profile update

BR adaptation determines the values of BR segments $\left\{\beta_{n}^{k+1}\right\}$ at iteration *k* + 1 based on $\left\{\beta_{n}^{k}\right\}$ and available glucose and insulin delivery information. The idea is to update the BR segment *β*_*n*_ with the averaged nonmeal‐related insulin microboluses commanded by the feedback controller during the same time interval, namely, $T_{n}^{\beta }$. Although different methods can be used to distinguish meal/nonmeal‐related insulin, we consider a simple approach in this work and define nonmeal‐related insulin as the insulin deliveries that happen *τ*_*m*_ hours after the previous meal. This defines a set of admissible meal‐related insulin deliveries $I_{n}^{d}$ for each BR segment *β*_*n*_:

(1) $$I_{n}^{d}≔\left\{\left(t,I_{t}^{d}\right)|t\in T_{n}^{\beta },t\geq T_{m}^{d}\left(t\right)+\tau_{m}\right\},$$

where $I_{t}^{d}$ denotes an insulin delivery at time instant *t* on day *d*, $T_{n}^{\beta }$ denotes the time period for which *β*_*n*_ is active, and $T_{m}^{d}\left(t\right)$ denotes the time of the previous meal that happens before time *t* on day *d*. We take *τ*_*m*_ = 3 hr in our implementation. An initial BR estimate ${\bar{\beta }}_{n}^{k}$ for $\beta_{n}^{k}$ can be written as

(2) $${\bar{\beta }}_{n}^{k}=\frac{\sum_{d=1}^{n_{w}}\sum_{t=1}^{n_{d}}I_{t}^{d}\cdot 1\left(\left(t,I_{t}^{d}\right)\in I_{n}^{d}\right)}{\sum_{d=1}^{n_{w}}\sum_{t=1}^{n_{d}}1\left(\left(t,I_{t}^{d}\right)\in I_{n}^{d}\right)},$$

where *n*_*d*_ denotes the number of insulin microboluses in a day, and *n*_*w*_ denotes the number of days in iteration *k* of the adaptation process, and **1**(⋅) denotes the indicator function. Although the actual real‐time BR will be decided by the feedback control algorithm (using the knowledge of *β*_*n*_), the key consideration on the obtained ${\bar{\beta }}_{n}$ is that it should not overestimate the “true” BR and cause hypoglycemic events, which is particularly important when the feedback controller is unavailable. To address this concern, two types of safety constraints are incorporated, as will be introduced below.

##### Statistical IOB constraint

Similar to the case with lower‐layer control algorithms, the IOB information can be exploited to prevent aggressive behavior of the parameter adaptation algorithm. On the basis of the IOB calculation used in zone MPC,^20^ a statistical IOB constraint is proposed for BR adaptation. Specifically, a dynamic database $D\left(\beta_{n}\right)$ is built to eliminate overestimated values of *β*_*n*_, which stores the information of a triplet $\left\{{\mathrm{IOB}}_{n}^{i},\beta_{n}^{i},\gamma_{m∼n}^{i}\right\}$ that have led to low‐glucose events in history for *i* ∈ {1, 2, …, *k*}, with ${\mathrm{IOB}}_{n}^{i}$ denoting the averaged IOB value immediately before *β*_*n*_ becomes active in adaptation iteration *i*, and $\gamma_{m∼n}^{k}$ denotes the values CR used within $T_{n}^{\beta }$ in iteration *k*. When ${\bar{\beta }}_{n}^{k+1}$ is computed for iteration *k* + 1 and ${\bar{\beta }}_{n}^{k+1}>\beta_{n}^{k}$, the IOB constraint is enforced and implemented as

(3) $$\left[{\mathrm{IOB}}_{n}^{k},{\bar{\beta }}_{n}^{k+1},1/\gamma_{m∼n}^{k}\right]\;⪯\;\left[{\mathrm{IOB}}_{n}^{i},0.95\beta_{n}^{i},1/\gamma_{m∼n}^{i}\right],$$

for all $\left\{{\mathrm{IOB}}_{n}^{i},\beta_{n}^{i},\gamma_{m∼n}^{i}\right\}\in D\left(\beta_{n}\right)$, where “⪯” denotes an element‐wise partial order holds for the two vectors compared. Here, ${\mathrm{IOB}}_{n}^{k}$ is used to estimate the average IOB value immediately before *β*_*n*_ becomes active in adaptation iteration *k* + 1. If any of the IOB constraints are violated, ${\bar{\beta }}_{n}^{k+1}=0.95\beta_{n}^{k}$ to avoid causing hypoglycemia risk.

##### Smoothness constraint

This constraint ensures that the BR segment *β*_*n*_ does not change too much compared with its neighboring segments $\beta_{n^{-}}$ and $\beta_{n^{+}}$, with *n*^−^ = *N* − mod(*N* − *n* + 1, *N*) and *n*^+^ = mod(*n*, *N*) + 1, respectively. The underlying intuition is that the insulin requirement of the metabolic system to maintain a healthy fasting glucose level does not change abruptly in time. Mathematically, this constraint is implemented as

(4) $$\beta_{n}^{k+1}=\min\left({\bar{\beta }}_{n}^{k+1},\lambda_{s}^{\beta }\min\left({\bar{\beta }}_{n^{-}}^{k+1},{\bar{\beta }}_{n^{+}}^{k+1},\beta_{n^{-}}^{k},\beta_{n^{+}}^{k}\right)\right),$$

where $\lambda_{s}^{\beta }$ denotes the “smoothness coefficient” and is selected as 1.3 in our work to compromise smoothness with performance.

#### 2.1.2 Adaptation of CR profile

Different from BR adaptation, the effect of different CR segments on glucose profiles are relatively isolated, as meals are usually several hours apart. This helps decouple the adaptation problems for different *γ*_*n*_ values, for which a data‐driven optimization approach can be developed.

##### Zone objectives

Instead of optimizing the CR profile for a specific glycemic metric, our goal for CR adaptation is to only ensure that the average BG levels $\left\{y_{n}^{\gamma }|n=1,2,\ldots ,M\right\}$ after meals are taken for *τ*_*γ*_ hours should settle within a certain zone $\left[{\bar{y}}^{\gamma },{\underline{y}}^{\gamma }\right]$; in our implementation, this zone is meal‐dependent and is selected as [125, 155], [135, 165], and [125, 155] mg/dl for breakfast, lunch, and dinner, respectively, based on simulation data from the UVA/Padova simulator. On one hand, this choice of a control‐to‐range objective helps efficiently handle the uncertainties caused by lifestyle disturbances (e.g., sizes and timing of meals). Conversely, as the final value of meal boluses are normally decided by the patient, the meal bolus sizes calculated using the CR profile are used as references, thus a robust estimate of CR would be more practical from an application perspective. In this work, *τ*_*γ*_ is selected to be 4 or the length of time elapsed before the next meal is taken if it is less than 4 hr, based on the observations of the UVA/Padova Simulator.

##### Parameter optimization with safety constraints

To adapt the CR profile toward the target zone, a virtual optimization problem is formulated for each *γ*_*n*_. The cost function is selected as the average postprandial BG levels

(5) $$y_{n}^{\gamma }\;≔\;f_{n}^{\gamma }\left(\gamma_{n},\theta_{n}^{\gamma }\right),$$

where $f_{n}^{\gamma }\left(\gamma_{n},\theta_{n}^{\gamma }\right)$ is used to represent the underlying unknown dependency of $y_{n}^{\gamma }$ on *γ*_*n*_ and other parameters $\theta_{n}^{\gamma }$. The adaptation process is then performed by solving a sequence of constrained optimization problems with this unknown cost function:

(6) $$\min_{\gamma_{n}^{k+1}}\;f_{n}^{\gamma }\left(\gamma_{n}^{k+1},\theta_{n}^{\gamma }\right)$$

(7) $$s.t.\;|\gamma_{n}^{k+1}-\gamma_{n}^{k}|\leq \lambda^{\gamma }\gamma_{n}^{k},$$

(8) $$\;|f_{n}^{\gamma }\left(\gamma_{n}^{k+1},\theta_{n}^{\gamma }\right)-f_{n}^{\gamma }\left(\gamma_{n}^{k},\theta_{n}^{\gamma }\right)|\leq \delta^{y_{n}^{\gamma }}$$

(9) $$\;\gamma_{n}^{k+1}\geq {\underline{\gamma }}_{n}^{k+1}.$$

To ensure the smoothness of the adaptation procedure, three safety constraints are considered in the optimization problem above. The first and second constraints, respectively, restrict the rate of change of *γ*_*n*_ and $f_{n}^{\gamma }\left(\gamma_{n},\theta_{n}^{\gamma }\right)$. In our implementation, *λ*^*γ*^ is chosen as 30%, and $\delta^{y_{n}^{\gamma }}$ is selected as 12 mg/dl. The third constraint directly bounds *γ*_*n*_ from below to avoid hypoglycemia risks caused by an underestimated CR; the lower bound ${\underline{\gamma }}_{n}^{k+1}$ is updated dynamically by taking the maximum value of $\gamma_{n}^{i}$ that has caused risk of hypoglycemia during the adaptation process. Note that $\gamma_{n}^{k}$ and ${\underline{\gamma }}_{n}^{k+1}$ are known and $f_{n}^{\gamma }\left(\gamma_{n}^{k},\theta_{n}^{\gamma }\right)$ can be calculated based on historical CGM measurements when $f_{n}^{\gamma }\left(\gamma_{n}^{k+1},\theta_{n}^{\gamma }\right)$ is optimized for $\gamma_{n}^{k+1}$, but the explicit expression of $f_{n}^{\gamma }\left(\gamma_{n}^{k+1},\theta_{n}^{\gamma }\right)$ is generally not known. To solve this problem, a data‐driven BO‐assisted algorithm will be provided in Section 2.3.

#### 2.1.3 Alternating BR and CR adaptation

**Figure 2:**
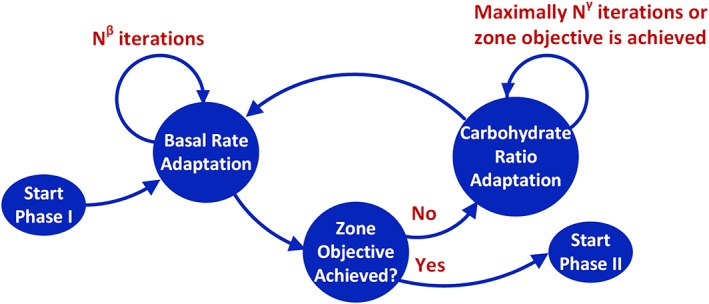
Adaptation of feedforward control parameters

Considering the coupling effects of BR and CR on glucose management, the adaptation of {*β*_*n*_| *n* = 1, 2, …, *N*} and {*γ*_*n*_| *n* = 1, 2, …, *M*} are performed in an interactive fashion. For safety consideration, only one profile (BR or CR) is adapted in each iteration. To do this, BR adaptation is performed in a time‐driven manner such that the values of {*β*_*n*_| *n* = 1, 2, …, *N*} are adapted for *N*^*β*^ consecutive iterations, which is set to 2 in our implementation. CR adaptation is performed in a combined time‐ and event‐triggered fashion; the adaptation procedure ends either when the zone objectives are achieved or when a maximum number of iterations *N*^*γ*^ is reached, which is set to 5 in our implementation. As meal boluses can be adjusted by the patients based on their knowledge and preference, the overall feedforward parameter adaptation phase begins by adapting the BR profile and iterates according to the alternating procedure described. An illustration of the procedure is provided in Figure 2. The phase terminates when the zone objective criterion for CR adaptation remains valid after performing the BR adaptation.

#### 2.1.4 Choice of parameters

A few parameters are adjustable in the proposed BR and CR adaptation procedure, including $\lambda^{\gamma },\;\delta^{y_{n}^{\gamma }},\;N^{\beta }$, and *N*^*γ*^, the values of which used in the in silico studies of this work have been provided in the above subsections. Specifically, *λ*^*γ*^ and $\delta^{y_{n}}$ are safety factors that limit the rates of change of the elements in the CR profile, while *N*^*β*^ and *N*^*γ*^ are the maximum allowable inner iterations to adapt BR and CR profiles (see also Figure 2) and thus jointly determine the duration of the adaptation process. With these physical interpretations, other values of these parameters can be selected based on clinical experience and knowledge of particular patients in clinical studies.

### 2.2 Learning feedback control parameters

In this subsection, we focus on Phase II of the adaptation procedure, which deals with parameter adaptation for feedback control. As the appropriately adjusted BR and CR profiles in Phase I provide optimized operating conditions for the closed‐loop controller, only moderate changes on the key parameters are needed to achieve satisfactory glucose regulation. Based on this observation, the approach here is to first determine the bottle‐neck parameter that limits the performance of closed‐loop control for a specific patient, and then to dynamically learn the appropriate value of the selected parameter. Ideally, improved performance could be potentially obtained by considering combined dynamic parameter selection and adaptation, but the improvement comes with compromised risk of hypoglycemia and time needed to complete the adaptation procedure.

#### 2.2.1 Parameter selection based on sensitivity analysis

Generally, parameter selection determines the parameter $\varphi \in \left\{\hat{R},\bar{D},\gamma_{\mathrm{IOB}}\right\}$ to be adapted in Phase II. In this work, a sensitivity analysis approach is utilized to achieve automatic parameter selection, by rerunning the closed‐loop control algorithm with different parameter settings using the most recent historical glucose measurements for a specific patient, which is the so‐called advisory‐mode analysis.^22^ To do this, for each $\varphi \in \left\{\hat{R},\bar{D},\gamma_{\mathrm{IOB}}\right\}$, a pair of upper and lower bounds {*φ*^+^, *φ*^−^} that limit the range of feasible choices of *φ* in the adaptation procedure are specified; in our implementation, {*φ*^+^, *φ*^−^} is chosen as {150%, 50%},  {122%, 89%}, and {120%, 70%} of the corresponding nominal values for $\hat{R},\;\bar{D},\;\text{and}\;\gamma_{\mathrm{IOB}}$, respectively. Advisory mode comparisons are performed by separately choosing $\varphi =\bar{\varphi }$ and $\varphi =\underline{\varphi }$ and keeping parameters in $\left\{\hat{R},\bar{D},\gamma_{\mathrm{IOB}}\right\}/\left\{\varphi \right\}$ unchanged. The sensitivity analysis is then performed by solving

(10) $$\varphi^{⋆}=\arg\min_{\varphi \in \left\{\hat{R},\bar{D},\;\gamma_{\mathrm{IOB}}\right\}}|I\left(\varphi^{+}\right)-I\left(\varphi^{-}\right)|,$$

where ℐ(*φ*^+^) and ℐ(*φ*^−^) denote the total amount of insulin calculated in the advisory mode analysis for *φ* = *φ*^+^ and *φ* = *φ*^−^, respectively. This procedure determines the parameter that has the “strongest” controllability of insulin delivery, hence the closed‐loop glycemic regulation can be adjusted most efficiently.

#### 2.2.2 Parameter optimization

The goal of adapting the selected parameter *φ* is to achieve satisfactory average glucose level without having risk of hypoglycemia, which implies improved percentage time in euglycemic range [70, 180] mg/dl as the glucose profile is restricted below by considering constraints on hypoglycemia. To achieve this goal, we again formulate the adaptation procedure as a sequential optimization problem:

(11) $$\min_{\varphi^{k+1}}\;f^{\varphi }\left(\varphi^{k+1},\theta^{\varphi }\right)$$

(12) $$s.t.\;|\varphi^{k+1}-\varphi^{k}|\leq \lambda^{\varphi }\varphi^{k},$$

(13) $$\;|f^{\varphi }\left(\varphi^{k+1},\theta^{\varphi }\right)-f^{\varphi }\left(\varphi^{k},\theta^{\varphi }\right)|\leq \delta^{y^{\varphi }},$$

(14) $$\max\left\{\varphi^{-},{\underline{\varphi }}^{k+1}\right\}\leq \varphi^{k+1}\leq \min\left\{\varphi^{+},{\bar{\varphi }}^{k+1}\right\}.$$

Here, *φ*^*k* + 1^ denotes the value of *φ* at the (*k* + 1)th iteration, *f*^*φ*^(*φ*^*k* + 1^, *θ*^*φ*^) denotes the average glucose value obtained using *φ*^*k* + 1^ with *θ*^*φ*^ being potential dependent parameters. The constraints in Equations 12 and 13 restrict the rate of change of *φ* and *f*^*φ*^(*φ*, *θ*^*φ*^), respectively. In our implementation, *λ*^*φ*^ is selected as 30%, and $\delta^{y^{\varphi }}$ is chosen as 6 mg/dl; the roles of these two parameters are identical to those of *λ*^*γ*^ and $\delta^{y_{n}^{\gamma }}$ discussed in Section 2.1.4. The constraints in Equation 14 bound the feasible region of *φ*; in addition to *φ*^−^ and $\varphi^{+},\;{\underline{\varphi }}^{k+1}$ and ${\bar{\varphi }}^{k+1}$ provide additional dynamic bounds based on historical values of *φ*, namely, {*φ*^1^, …, *φ*^*k*^}, to help avoid hypoglycemia risks. The sequential optimization procedure ends either when average glucose level becomes satisfactory (less than 135 mg/dl in our implementation) or when the bounds in Equation 14 become active, the latter of which means the controller has achieved its performance limitation. Similar to the case of adapting *γ*_*n*_, the analytical expression of the cost function *f*^*φ*^(*φ*^*k* + 1^, *θ*^*φ*^) is not known. A BO‐assisted optimization algorithm will be introduced to solve this problem.

### 2.3 BO‐assisted parameter adaptation

In Sections 2.1.2 and 2.2.2, optimization problems with unknown objective functions and constraints need to be solved. Specifically, the two problems in Equations 6‐9 and 11‐14 share the form

(15) $$\min_{\psi^{k}}\;f\left(\psi^{k},\theta^{\psi }\right)$$

(16) $$s.t.,\;|f\left(\psi^{k},\theta^{\psi }\right)-y_{k-1}|\leq \delta ,$$

(17) $$\;g\left(\psi^{k}\right)\leq 0,$$

where *f*(*ψ*^*k*^, *θ*^*ψ*^) is an unknown function of *ψ*^*k*^ parameterized by *θ*^*ψ*^, *y*_*k* − 1_ is a noisy measurement of *f*(*ψ*^*k* − 1^, *θ*^*ψ*^), *δ* is a known parameter, and *g*(*ψ*^*k*^) is a known (linear) function of *ψ*^*k*^. Noticing the fact that a noisy measurement/estimate *y*_*k*_ of *f*(*ψ*^*k*^, *θ*^*ψ*^), which is average glucose level for a fixed *ψ* ^*k*^, is available, we solve this problem through designing a BO‐assisted optimization algorithm, to make efficient use of historical measurements of {*f*(*ψ*^*k*^, *θ*^*ψ*^)| *k* ∈ *π*_*k*_} for an admissible set of iteration indexes *π*_*k*_; here, *π*_*k*_ is defined as the set of iteration indexes for which the same set of parameters are adapted while other adaptation parameters are kept constant. The main idea is to obtain a data‐driven estimate $\hat{f}\left(\psi_{k},\theta_{k}|D_{k}\right)$ of *f*(*ψ*^*k*^, *θ*^*ψ*^) based on available historical data $D_{k}$. In the BO literature, different forms of $\hat{f}\left(\psi_{k},\theta_{k}|D_{k}\right)$ have been proposed.^23^ As the size of data set for adaptation is small compared with those available for machine learning problems, a linear kernel is adopted in this work:

(18) $$\hat{f}\left(\psi^{k},\theta_{k}|D_{k}\right)≔\theta_{k,1}\psi^{k}+\theta_{k,2}$$

with *θ*_*k*_ ≔ [*θ*_*k*, 1_, *θ*_*k*, 2_]^⊤^. Note that to make efficient use of data, the value of *θ*_*k*_ is made *ψ*‐dependent and is obtained based on data segment ${\bar{D}}_{k}\subseteq D_{k}$, which is the subset of data $D_{k}$ corresponding to iteration indexes in *π*_*k*_. This further explains the rationale for using a linear kernel: the cost function in Equation 18 simply represents a local linearization of the unknown cost function *f*(*ψ*^*k*^, *θ*^*ψ*^) around the adapted values of *ψ* ^*k*^.

To solve Equations 15‐17, a BO‐based algorithm is proposed (see Algorithm 1), which iteratively adapts tuning parameter *ψ*_*k*_ until the problem‐dependent terminal conditions are satisfied. For each iteration, the algorithm starts with updating the admissible set *π*_*k*_, and obtains ${\bar{D}}_{k}\subseteq D_{k}$ based on *π*_*k*_ (line 2 of the algorithm). For the problems in Equations 6‐9 and 11‐14, the effect of the adaptation parameter *ψ* on *f*(*ψ*^*k*^, *θ*^*ψ*^) (which can be understood as the sign of the partial derivative) is known, and can be exploited to determine the search direction $S_{k}$ (line 3); this helps ensure that the algorithm can evolve along the correct direction with noisy measurements {*y*_*k*_}. If there is not enough data to perform data fitting (line 4), heuristic adjustment of *φ*_*k*_ will be performed along $S_{k}$ for safety concerns, where $\delta^{S}$ is selected as 10% in our implementation. For all other scenarios, *φ*_*k*_ is adjusted through the proposed BO procedure (lines 5–11). The BO first estimates *θ*_*k*_ based on ${\bar{D}}_{k}$ (line 6). With the obtained *θ*_*k*_, *ψ*_*k*_ is calculated by solving a constrained optimization problem (line 7). As $\hat{f}\left(\varphi_{k},\theta_{k}|D_{k}\right)$ is linear with respect to *φ*_*k*_, the optimization problem is actually a linear programming problem. To enhance the robustness of the algorithm against noises in {*y*_*k*_}, the deviation of *ψ*_*k*_ from *ψ*_*k* − 1_ is compared with $S_{k}$; the value of *ψ*_*k*_ will be recalculated if inconsistency is observed (line 8). Safety checks are further performed for *ψ*_*k*_ (line 10), where the value of *ψ*_*k*_ is truncated if the constraint is violated. The obtained *φ*_*k*_ is then implemented to obtain *y*_*k*_, which is collected to update $D_{k}$ (line 12).

## 3 RESULTS

In this section, we evaluate the proposed adaptation algorithm through multiple‐month simulations on the 111‐patient cohort of the US FDA accepted UVA/Padova simulator.^16^ To mimic intra‐day variations in insulin sensitivity and the effect of dawn phenomenon, the diurnal patterns of the parameters in the endogenous glucose production and glucose utilization models are implemented according to Reference, 24 Inter‐day variation in insulin sensitivity is further introduced by adding random noise obeying a uniform distribution within ±5% of the nominal values of the parameters that determine insulin sensitivity and dawn phenomenon. To consider the effect of illness on insulin sensitivity, a healthy in silico patient has a 5% chance of entering a sick state that can last up to five consecutive days; when an illness event happens, it can either increase by 50% or decrease by 100% the magnitudes of the insulin sensitivity and dawn phenomenon parameters with probabilities of 0.5, throughout the illness period. We assume that the state of sickness is not announced to the adaptation algorithm. To mimic lifestyle disturbances, the in silico subjects take breakfast, lunch, and dinner with normally distributed meal sizes (with means and standard deviations equal to [50, 75, 75] g and [3, 4, 4] g carbohydrate [CHO]) and meal times uniformly distributed in [07:00, 09:00], [11:00, 13:00], and [18:00, 20:00], respectively; in addition, each meal can be skipped with probability 0.1. The CGM measurement noise is generated according to a random noise seed on each day. We assume that the meals are all fully announced but the meal boluses are calculated with potentially inappropriate CR profiles. The updated parameters obtained in each iteration are used for 1 week (7 days)^10^ before the next iteration, so that enough data can be collected for performance evaluation. For implementation purpose, we assume the BR profile contains five segments, the effective period of which are [02:00, 05:00], [05:00, 10:00], [10:00, 16:00], [16:00, 21:00], and [21:00, 02:00], respectively; the CR profile are composed of four segments, the effective period of which are [05:00, 10:00], [10:00, 16:00], [16:00, 21:00], and [21:00, 05:00], respectively; only the first three segments that are responsible to breakfast (B), lunch (L), and dinner (D) are adapted and the segment that effective overnight is set to the default value from the simulator.

To evaluate the safety, effectiveness, and robustness of the adaptation algorithm, three scenarios are considered. In the first scenario (Scenario I), the patients are assumed to have doubled CR and halved BR profile segments compared with the default values in the simulator; both of these settings will lead to increased hyperglycemia due to conservative insulin delivery. In the second scenario (Scenario II), the patients are initiated with doubled CR and doubled BR profiles; the former would cause conservative meal boluses but the latter would counteract with relatively larger insulin microboluses, which makes it challenging for the adaptation algorithm to identify the appropriate tuning parameters. The third scenario (Scenario III) mimics real‐life situations, in which different segments in the BR profile and CR profile may deviate within a reasonable range from either above or below the corresponding appropriate values; to do this, the in silico subjects are initialized with randomized BR and CR profiles, in which the segments are generated according to uniform distributions within [50%, 150%] of the corresponding default values. For all in silico subjects, we assume the zone MPC developed in Reference 20 with default parameters is used. The parameters in the adaptation algorithm are specified in Sections 2.1–2.3. For all three scenarios, the first week is utilized to collect data for initialization and thus no parameter is adjusted; the adaptation process starts from the second week. All simulations are run for 24 weeks. The key glycemic metrics obtained before and after the proposed adaptation algorithm are provided in Table 1.

**Table 1:** Glycemic metrics obtained before and after the proposed adaptation algorithm

	Scenario I			Scenario II			Scenario III		
Metrics	Week 1	Week 24	*p* value	Week 1	Week 24	*p* value	Week 1	Week 24	*p* value
Percentage time < 54 mg/dl	0.0 (0.0)	**0.0 (0.0)**	.017*	7.0 (7.6)	**0.0 (0.0)**	<.001*	0.0 (0.0)	0.0 (0.0)	.763
Percentage time < 70 mg/dl	0.0 (0.0)	**0.0 (0.4)**	<.001*	15.7 (8.0)	**0.0 (0.1)**	<.001*	0.1 (1.0)	0.0 (0.2)	.077
Percentage time 70–180 mg/dl	41.1 (18.2)	**88.1 (9.5)**	<.001*	75.6 (9.9)	**88.8 (10.2)**	<.001*	81.6 (12.4)	**88.7 (13.8)**	<.001*
Mean BG (mg/dl)	194.3 (15.9)	**142.3 (8.8)**	<.001*	110.5 (10.8)	**142.0 (8.9)**	<.001*	144.3 (14.2)	142.4 (9.1)	.756

Data in this table are shown as median (inter quartile range). Statistical significance is assessed by Wilcoxon signed‐rank test. Statistically significant (*p* < .05) changes are highlighted in bold with asterisks.

### 3.1 Results for Scenario I

**Figure 3:**
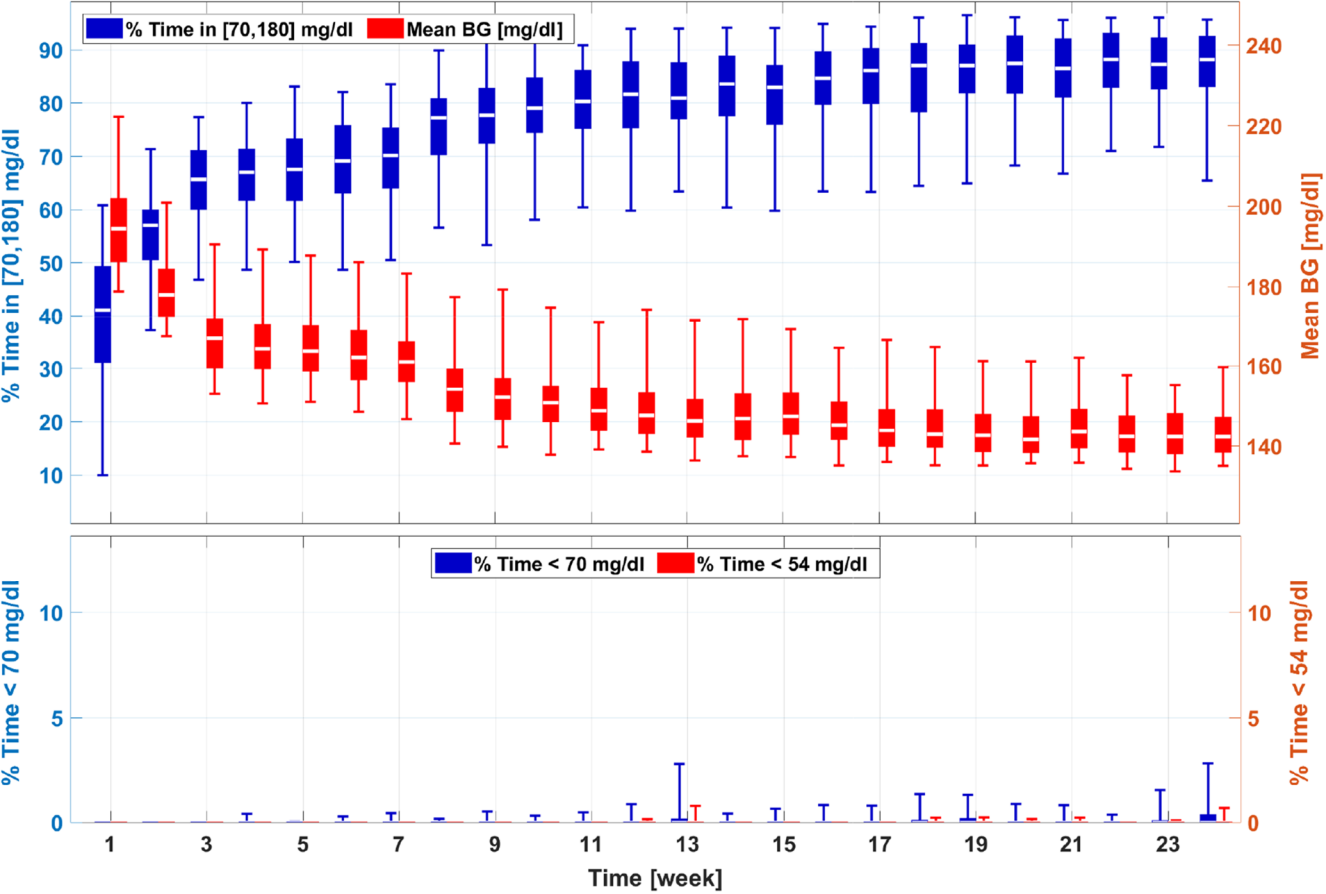
Trends of the glycemic management metrics in the adaptation procedures for the 111‐patient cohort (Scenario I). A box‐and‐whisker approach is used to plot the data, where on each box, the central white line is the median, the edges of a box denote the 25% and 75% percentiles, and the whiskers denote the 5% and 95% percentiles

**Figure 4:**
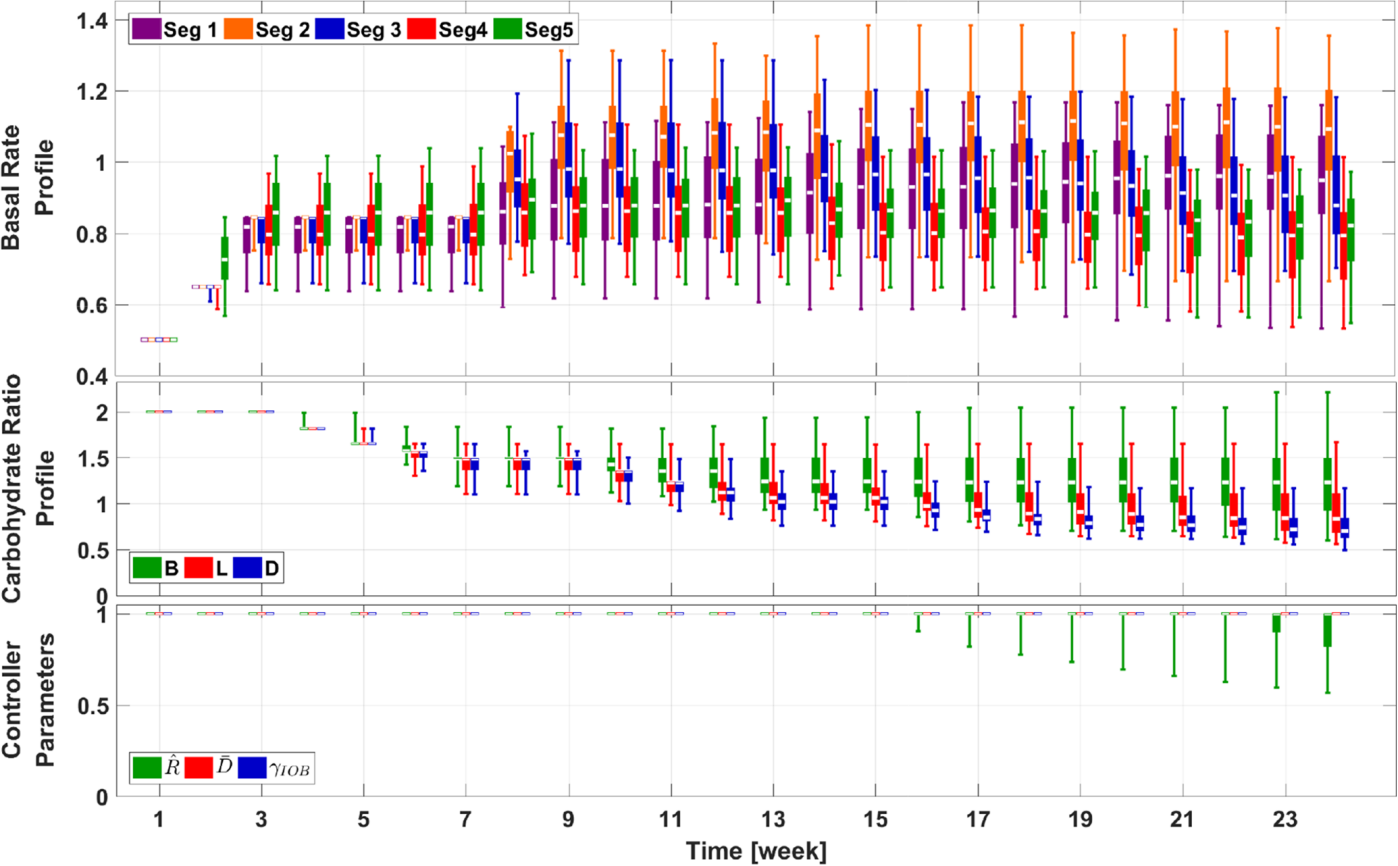
Trends of the adaptation parameters in the adaptation procedures for the 111‐patient cohort (Scenario I). The same box‐and‐whisker approach as that in Figure 3 is used to plot the data. For illustration purpose, the relative values of parameters against default values in the UVA/Padova simulator are provided

The results for Scenario I are shown in Figures 3 and 4. Figure 3 provides the trends of key glycemic metrics during the adaptation procedure, including average BG levels, percentage time in the euglycemic range [70, 180] mg/dl, percentage time below 70 mg/dl, and percentage time below 54 mg/dl. Due to the joint effect of underestimated BR and overestimated CR profiles, the in silico subjects have elevated glucose levels on Week 1. From Week 2, monotonic and steady improvements are achieved by the proposed adaptation algorithm, and a trend of convergence in the performance metrics is observed around Week 12 as no significant changes happened in the rest of the simulations. For the 111‐subject cohort, the adaptation algorithm manages to improve glycemic control performance dramatically in terms of both average glucose levels (from 194.3 mg/dl [Week 1] to 142.3 mg/dl [Week 24]; *p* < .001) and average percent time in euglycemia range [70, 180] mg/dl (from 41.0% to 88.1%; *p* < .001). The adaptation algorithm is safe in the sense that no hypoglycemia risk is caused during the process.

The trends of parameter changes during the adaptation procedure are provided in Figure 4. The important observation here is that through the use of statistical IOB constraints and smoothness constraints, strong performance is achieved by the BR profiles with their segments aligning around the reference value (which is 1 in Figure 4), instead of profiles with extremely large and small neighboring segments. In addition, the CR profile segments are aligned around the reference value; due to the safety constraints, risky (small) values for the CR segments are avoided. In addition, only moderate changes are observed for parameters in the feedback controller, which is expected as the obtained feedforward control parameters (BR and CR profiles) have set up an optimized operating point for the feedback controller, and thus the default values can achieve satisfactory glucose management. It is interesting to note that the second segment of the BR profile is larger than the other elements, which helps counteract against diurnal insulin sensitivity changes and dawn phenomenon.

### 3.2 Results for Scenario II

**Figure 5:**
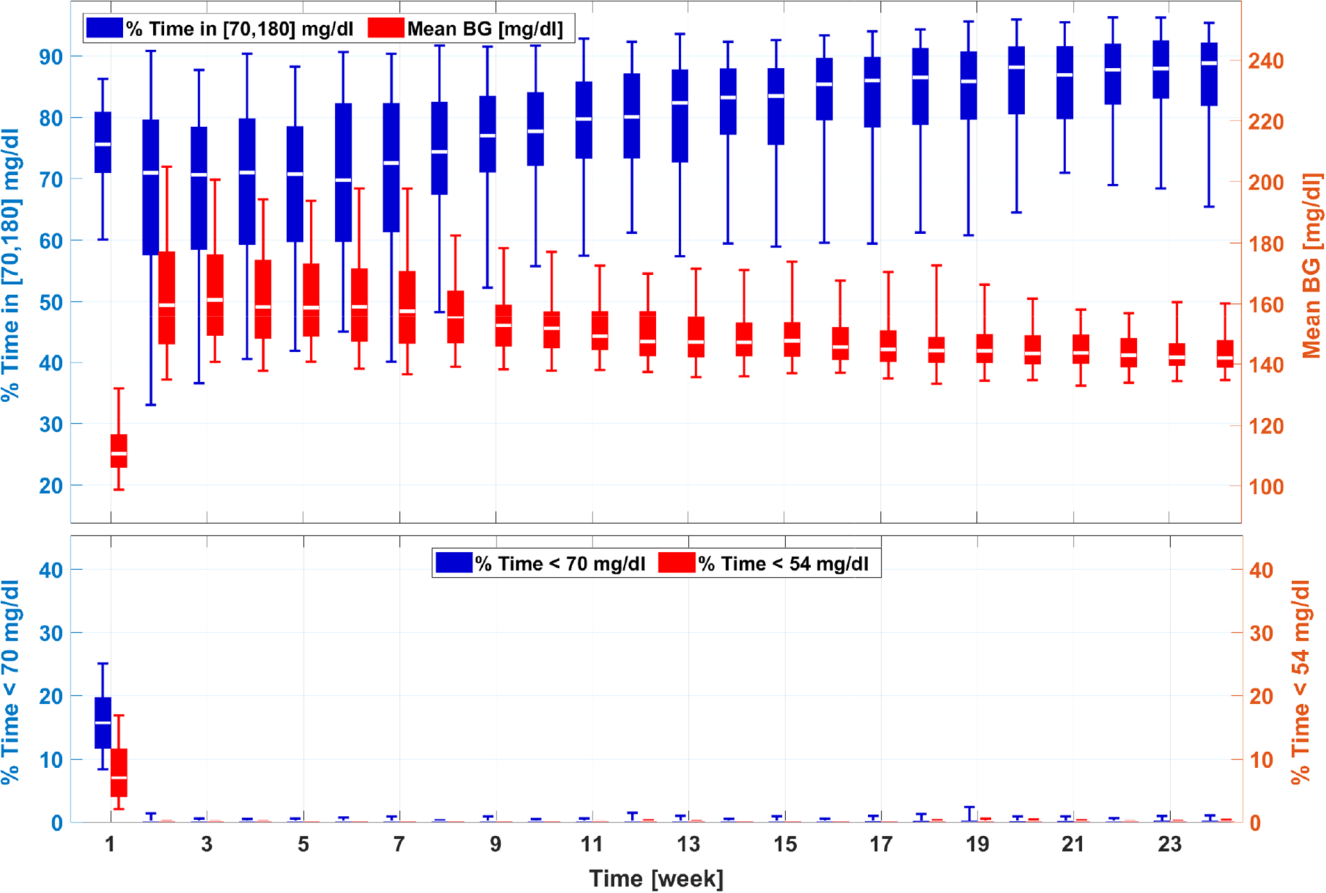
Trends of the glycemic management metrics in the adaptation procedures for the 111‐patient cohort (Scenario II). The same box‐and‐whisker approach as that in Figure 3 is used to plot the data

**Figure 6:**
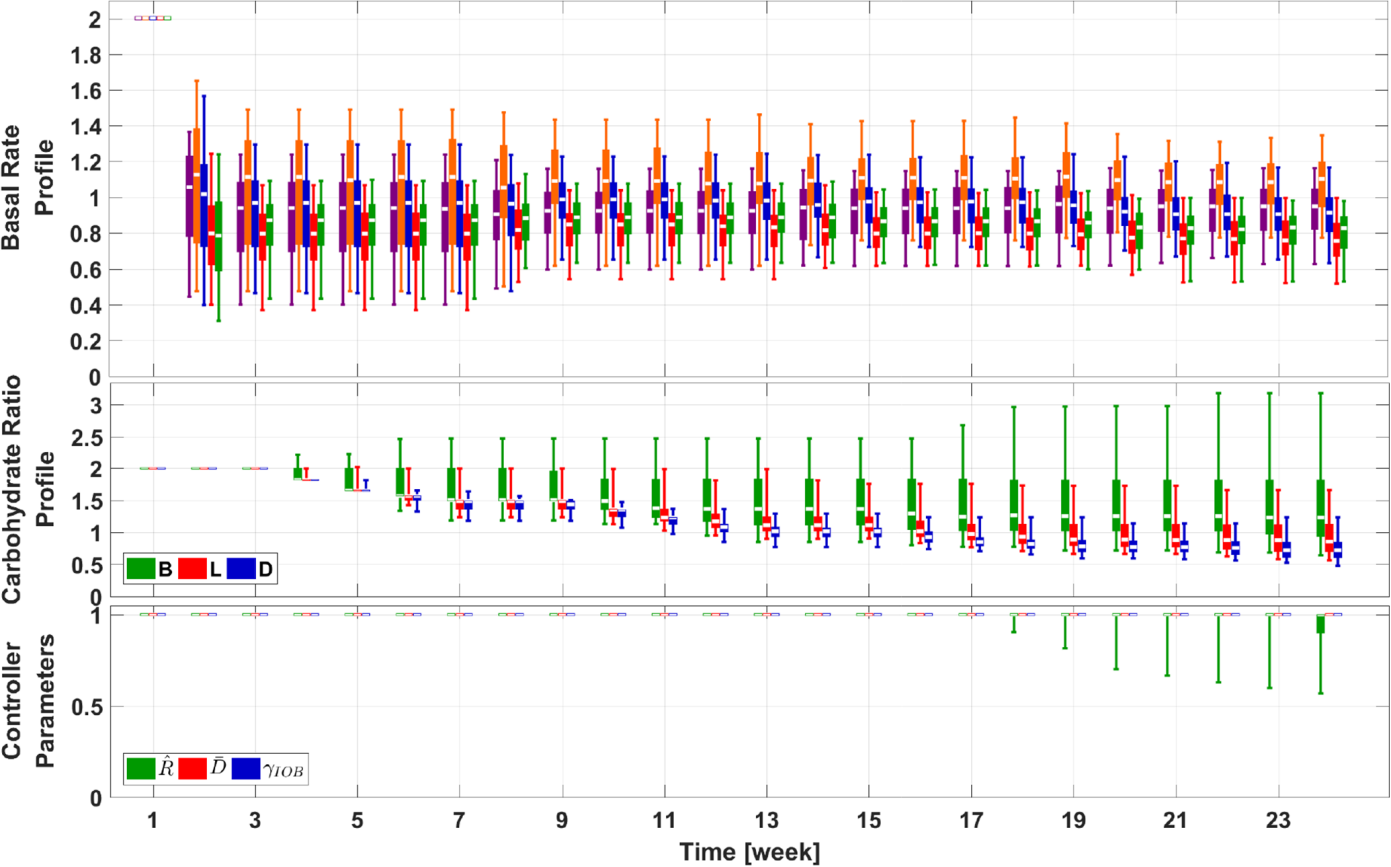
Trends of the adaptation parameters in the adaptation procedures for the 111‐patient cohort (Scenario II). The same box‐and‐whisker approach as that in Figure 3 is used to plot the data. Keys are the same as those in Figure 4

The results for Scenario II are provided in Figure 5 and 6. Due to the increased BRs, severe hypoglycemia is observed on Week 1 of the simulation. The controller‐led BR adaptation algorithm, however, is able to recognize safe BR profiles for the in silico cohort from Week 2 and perform safe fine tunes for the rest of the adaptation procedure. The CR adaptation procedure works properly as well, which manages to decrease the segments in the CR profiles for improved glycemic metrics. By eliminating risks of hypoglycemia (average percent time below 70 mg/dl, from 15.7% [Week 1] to 0.0% [Week 24], *p* < .001), the adaptation algorithm improves average percent time in [70, 180] mg/dl (from 75.6% [Week 1] to 88.8% [Week 24], *p* < .001). The obtained patterns of the BR profile and CR profile are similar to that of Scenario I.

### 3.3 Results for Scenario III

**Figure 7:**
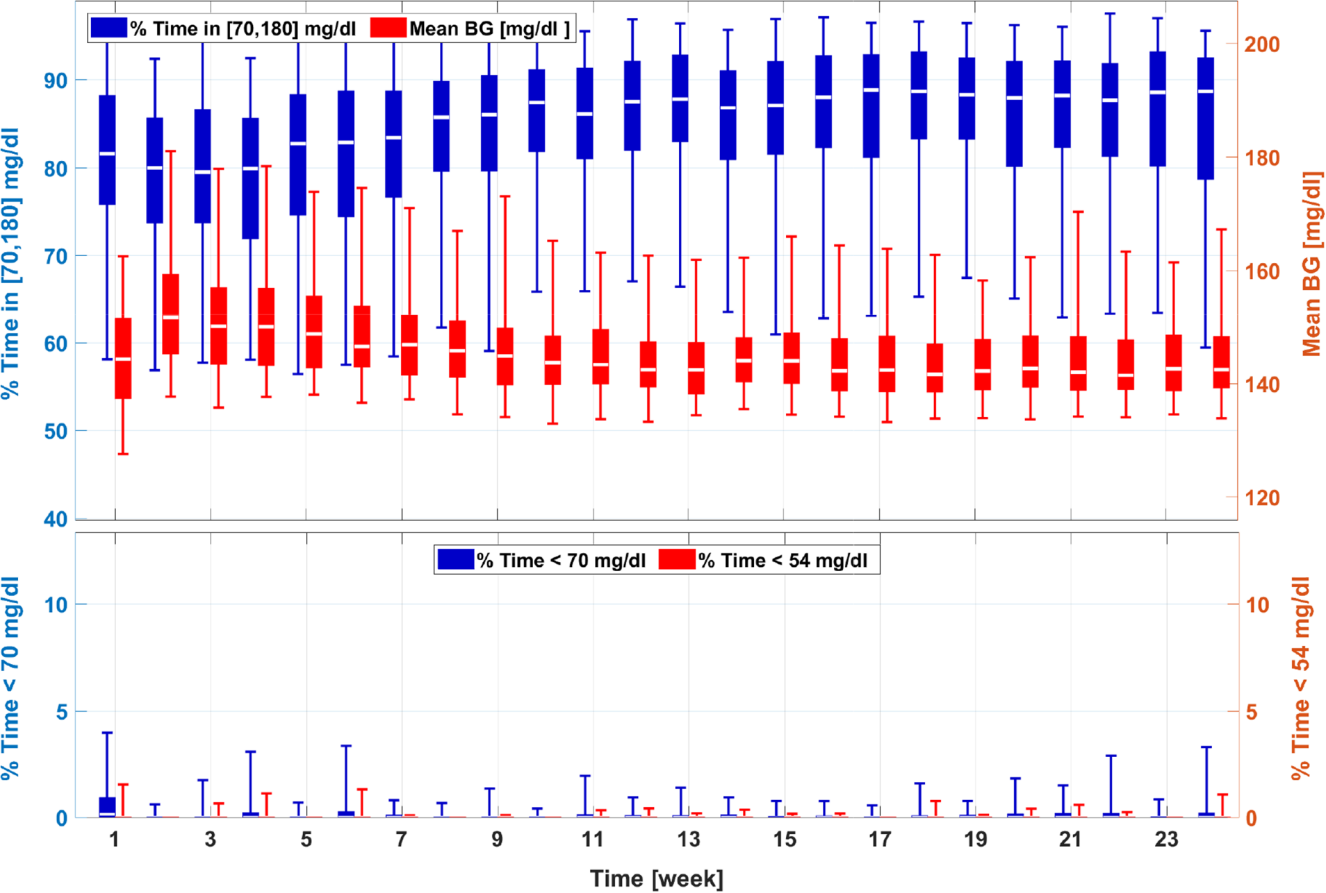
Trends of the glycemic management metrics in the adaptation procedures for the 111‐patient cohort (Scenario III). The same box‐and‐whisker approach as that in Figure 3 is used to plot the data

**Figure 8:**
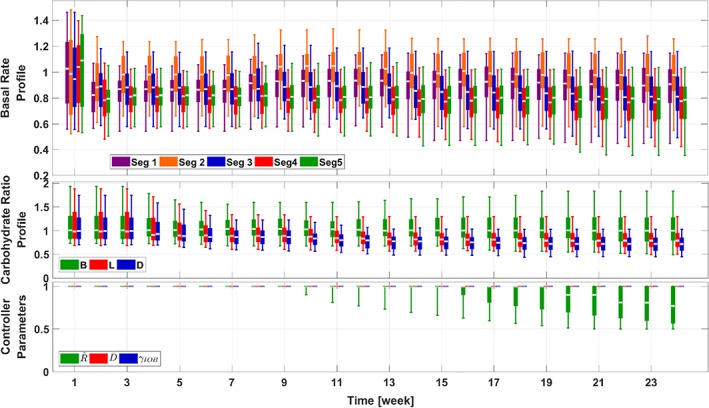
Trends of the adaptation parameters in the adaptation procedures for the 111‐patient cohort (Scenario III). The same box‐and‐whisker approach as that in Figure 3 is used to plot the data. Keys are the same as those in Figure 4

This scenario is devoted to simulate a 6‐month study of AP parameter adaptation, for patients with mixed and reasonably over‐ or underestimated segments in their BR and CR profiles. The results are provided in Figures 7 and 8. As expected, with slightly incorrect BR and CR profiles, the in silico patients generally have acceptable glucose management performance, which challenges the proposed adaptation method on the ability of recognizing and adjusting small but realistic mismatches in the adaptation parameters. From both figures, we observe that the algorithm is able to discern these parameter mismatches and achieve improved average percentage time in [70, 180] mg/dl (from 81.6% on Week 1 to 88.7% on Week 24), *p* < .001, without causing risk of hypoglycemia. It appears that average percentage time below 70 mg/dl is decreased as well, but the significance is not sufficiently small (from 0.1% to 0.0%, *p* = .077). Similar patterns in the BR and CR profiles to that obtained in the first two scenarios are realized.

### 3.4 Intra‐patient changes

**Figure 9:**
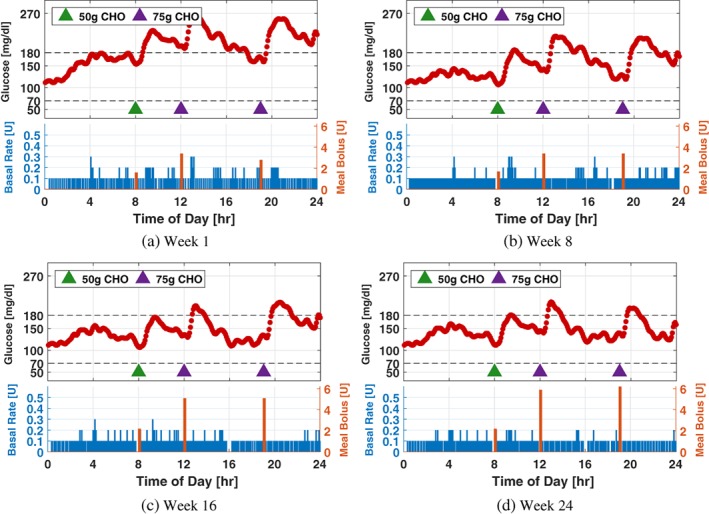
Twenty‐four‐hour glucose and insulin profiles simulated using the system parameters obtained on Weeks 1, 8, 16, and 24 of Scenario I for a particular patient. For comparison purpose, the same meal protocol and measurement noise sequence are applied to generate the data in the four subplots. Green and purple triangles denote meals of 50 g and 75 g CHO, respectively

**Figure 10:**
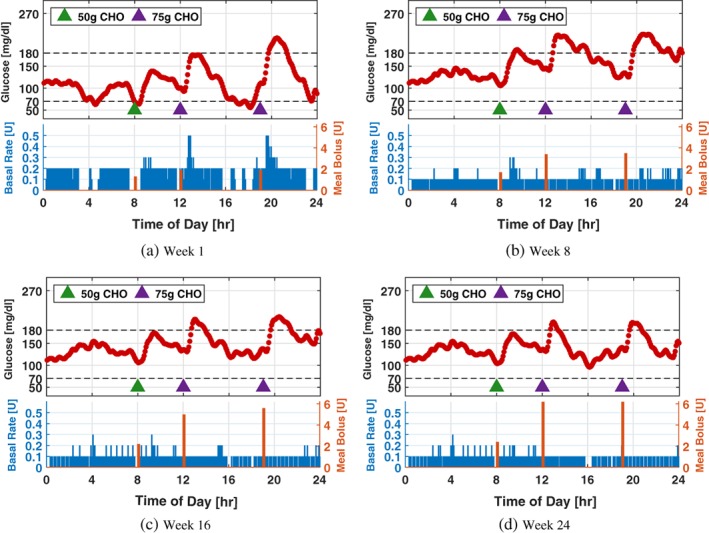
Twenty‐four‐hour glucose and insulin profiles simulated using the system parameters obtained on Weeks 1, 8, 16, and 24 of Scenario II for a particular patient. Meal protocol and sensor noise sequence are the same as those in Figure 9

**Figure 11:**
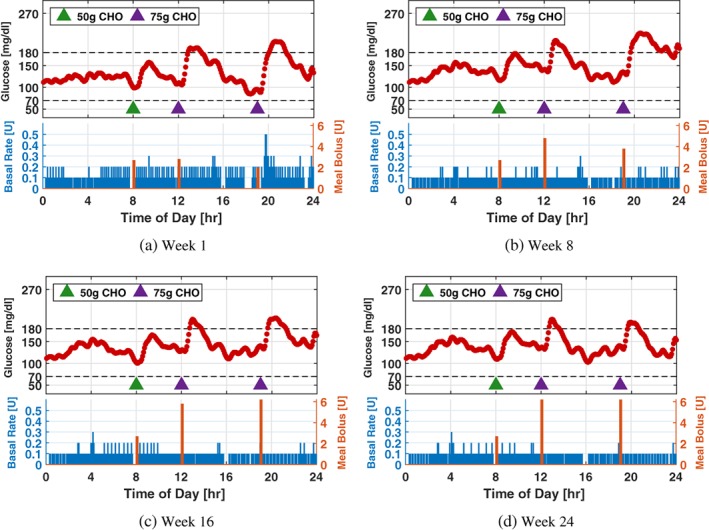
Twenty‐four‐hour glucose and insulin profiles simulated using the system parameters obtained on Weeks 1, 8, 16, and 24 of Scenario III for a particular patient. Meal protocol and sensor noise sequence are the same as those in Figure 9

To further illustrate the effect of the adaptation process, glucose and insulin profiles of a particular patient simulated using the adaptation parameters obtained on Weeks 1, 8, 16, and 24 are provided in Figure 9 (Scenario I), Figure 10 (Scenario II), and Figure 11 (Scenario III), in which a 24‐hr protocol composed of a 50 g CHO breakfast at 08:00, a 75 g CHO lunch at 12:00, and a 75 g CHO dinner at 19:00 with the sensor noise seed is used. The results obtained are consistent with the population‐level analysis in this section.

## 4 DISCUSSION

Several challenges exist in approaching the AP parameter adaptation problem. First, the effect of different AP parameters on glucose management is usually strongly coupled. For instance, the size of a real‐time insulin microbolus is jointly determined by the BR profile and the closed‐loop feedback controller, while BR, feedback controller, and user‐requested meal boluses can all contribute to meal‐related insulin delivery. This causes loss of identifiability to determine the correct root cause of poor glucose management; adjusting an incorrect parameter, however, may cause disastrous consequences. Second, lifestyle disturbances can also mask issues in glucose management. For instance, if a patient had a low BR for some time period while he/she happened to be jogging (which would increase insulin sensitivity) at the same time, the glucose traces might still appear to be acceptable, which would prevent an adaptation algorithm from identifying the real problems in glucose regulation. Finally, a considerable number of parameters in AP need to be considered as candidate variables in the adaptation procedure (which is 12 in our in silico tests in this work), while from a user experience perspective, the time of adaptation is limited (e.g., 24 weeks). As an iteration of the adaptation procedure may take 1 week to average out lifestyle disturbances, this basically implies that the task of AP adaptation through adjusting 12 coupled parameters need to be achieved within 24 iterations.

To overcome these challenges, a data‐driven multivariate learning framework is developed in this work, on the basis of a dual‐layer control scheme for long‐term home use of AP. In this scheme, feedback/feedforward control algorithms operate in the lower layer to achieve real‐time glucose regulation, while the adaptation algorithm is implemented in the upper layer based on data from the lower layer. The proposed adaptation procedure is composed of two phases. The first phase focuses on adaptation of feedforward control parameters, including BR and CR profiles. To bypass the nonidentifiability issue, a controller‐led approach is proposed for BR adaptation; the key idea is to exploit the intelligence from lower‐layer feedback control algorithm to achieve autonomous decision of BR profiles. IOB and smoothness constraints are proposed to avoid hypoglycemia risks in the controller‐led decision procedure. The CR profile is adapted through optimizing the postprandial BG levels toward a user‐specified target zone while considering dynamically updated data‐driven safety constraints. A hybrid time‐ and event‐triggered iterating procedure is proposed to achieve joint adaptation of BR and CR profiles. The second phase is devoted to adapting feedback control behavior. To improve the efficiency of adaptation, a sensitivity analysis is proposed through performing advisory mode comparison based on historical glucose data,^22^ so that the bottleneck control parameter can be selected for adaptation. The selected parameter is then updated by optimizing average glucose levels while restricting risks of hypoglycemia.

For optimization problems encountered in CR profile and feedback control adaptation, analytical relationships between optimization parameters and performance metrics are generally not known, which precludes the use of standard optimization methods.^25^ To overcome this additional challenge, the optimization problems are solved utilizing a BO approach, which was developed to optimize unknown objective functions based on noisy measurements in the machine learning community,^23^ and has been recently used in on‐line dynamics learning, automatic controller tuning and nonlinear adaptive control.^26^, ^27^, ^28^, ^29^ In our work, we integrate BO with safety requirements and clinical experience, so that the “black‐box” glucose regulation process can be safely adapted and improved.

The proposed adaptation method is evaluated on the basis of the 111‐patient cohort of the U.S. FDA accepted UVA/Padova simulator^16^ for three in silico scenarios. The first two scenarios focus on edge cases with (a) underestimated BR and overestimated CR profiles and (b) overestimated BR and CR profiles, while the third scenario considers a real‐life situation that different segments in the BR and CR profiles can be either overestimated or underestimated within a moderate extent. To challenge the proposed algorithm, lifestyle disturbances caused by timing and size changes for meals, inter‐ and intra‐day insulin sensitivity changes, and occasionally occurring sickness that can significantly offset insulin sensitivity. We show that for all scenarios, the proposed method is able to correctly identify and adaptively adjust the inappropriate parameters to achieve improved and satisfactory glucose regulation, without causing risks of hypoglycemia throughout the adaptation procedure.

## 5 CONCLUSION

In this article, a data‐driven multivariate learning approach has been proposed for long‐term parameter adaptation of an AP, on the basis of a dual‐layer control scheme. Through a two‐phase adaptation procedure, the algorithm gradually adjusts BR profile, CR profile, and parameters in the closed‐loop control algorithm. The adaptation of BR profile was performed by based on the behavior of the closed‐loop control algorithm while considering IOB and smoothness constraints, and CR profile and feedback control parameters were adjusted through a BO‐assisted approach. It was shown that the algorithm was able to adjust inappropriate parameters for improved glucose regulation, despite of lifestyle disturbances caused by skipped meals, sickness, and inter‐ and intra‐day insulin sensitivity changes.

## CONFLICT OF INTERESTS

The authors have no conflicts of interest to declare.

Algorithm 1 BO‐assisted parameter adaptation

1: **while** termination conditions not satisfied **do.**

2: update iteration counter *k* ≔ *k* + 1, update *π*_*k*_ and query $D_{k}$ for data ${\bar{D}}_{k}$ used in adaptation of *ψ*;

3: update $S_{k}$ based on *y*_*k* − 1_;

4: **if** ∣*π*_*k*_ ∣  ≤ *n*_BO_ **then.**

$\psi_{k}=\left(1+\delta_{s}S_{k}\right)\psi_{k-1};$

5: **else.**

6: update *θ*_*k*_ through solving.

$\;\min_{\theta }\sum_{k\in \pi_{k}}{\left(y_{k}-\hat{f}\left(\psi_{k},\theta |D_{k}\right)\right)}^{2};$

7: obtain *ψ*_*k*_ by solving.

$\;\min_{\psi_{k}}\;\hat{f}\left(\psi_{k},\theta_{k}|D_{k}\right)$

$s.t.,\;|\hat{f}\left(\psi_{k},\theta_{k}|D_{k}\right)-y_{k-1}|\leq \delta ;$

8: **if** sign(*ψ*_*k*_ − *ψ*_*k* − 1_) is inconsistent with $S_{k}$ **then.**

$\psi_{k}=\left(1+\delta_{s}S_{k}\right)\psi_{k-1};$

9: **end if.**

10: update *ψ*_*k*_ through safe checks.

$\hat{g}\left(\psi_{k}|D_{k}\right)\leq 0;$

11: **end if.**

12: implement *ψ*_*k*_ to obtain *y*_*k*_, and update data set $D_{k+1}≔D_{k}\cup \left\{\Delta_{k}\right\}$ with Δ_*k*_ ≔ {*y*_*k*_, *ψ*_*k*_};

13: end while.
